# Registered nurses’ experiences regarding clinical virtual learning during COVID-19 in Gauteng

**DOI:** 10.4102/hsag.v30i0.3118

**Published:** 2025-11-05

**Authors:** Neliswa L. Simelane, Nombulelo V. Sepeng, Kapari Mashao

**Affiliations:** 1 Department of Nursing Science, Faculty of Health Sciences, University of Pretoria, Pretoria, South Africa

**Keywords:** experiences, impact, professional practice, newly registered nurses’ competency, transition, virtual clinical learning, COVID-19

## Abstract

**Background:**

Virtual clinical learning variedly impacted newly registered nurses’ confidence. For some, it led to a lack of confidence in performing specific procedures, while for others, effective virtual learning fostered a high confidence level. This dynamic is likely to be even more complex when newly qualified nurses transition from education to practice, as healthcare facilities have high expectations, anticipating a certain level of knowledge and competence despite the challenges posed by coronavirus disease 2019 (COVID-19).

**Aim:**

The study explored newly registered nurses’ virtual experiences of learning clinical skills during COVID-19 and its impact on their transition into professional practice in the Tshwane District of Gauteng province in South Africa.

**Setting:**

The study was conducted in two selected hospitals in the Tshwane District of Gauteng province.

**Methods:**

Using unstructured interviews, a qualitative exploratory, descriptive and contextual design was executed to collect data. Purposive and snowballing sampling were utilised to reach to the sample of 12 registered nurses.

**Results:**

Three themes emerged in this study, namely: (1) Impact of virtual learning on confidence in clinical practice, (2) Challenges of transitioning from virtual learning to practical application and (3) Recommendations for enhancing virtual learning in nursing education.

**Conclusion:**

The study revealed both potential benefits and drawbacks of virtual clinical teaching on registered nurses’ transition into professional practice.

**Contribution:**

The findings may guide strategies to help nursing education institutions design virtual learning that ensures competent, practice ready nurses.

## Introduction

Theoretical and practical integration is crucial to improve comprehension and care-based clinical teaching experience (NDoH 2022). Developing clinical abilities is fundamental to nursing students’ undergraduate education (Barisone et al. 2019). Prior to coronavirus disease 2019 (COVID-19), nursing students were taught clinical skills in simulation laboratories for skills induction using low, medium, high fidelity and standardised patients (Koukourikos et al. 2021). Koukourikos et al. (2021) state that afterwards, they were allocated to clinical settings to integrate what they had learned in simulation laboratories into real-life clinical practices. Coronavirus disease 2019 forced a swift and extraordinary shift to online learning in several academic subjects, affecting professions such as nursing that significantly depend on hands-on training (Haanes et al. 2024). Gause, Mokgaola and Rakhudu (2022) state that during the COVID-19 shutdown, many educational institutions found adjusting to a virtual approach to learning extremely difficult. Clinical virtual simulation involves actual individuals with simulated systems to recreate reality as it appears on a computer screen (Padilha et al. 2019). Applications such as virtual reality and virtual patients are considered practical, speed up acquiring new skills and provide a stress-free learning environment (Chang & Lai 2021). Purwanti et al. (2022) indicate that one of the advantages is that virtual simulations effectively enhance students’ clinical nursing education skills. Purwanti et al. (2022) indicate that students who use virtual applications to play outperform those who do not in terms of clinical reasoning. However, Barisone et al. (2019) state that it can occasionally be challenging for nurse educators to discover pertinent audio-visual resources to aid in acquiring these clinical skills.

Among the difficulties encountered while utilising technology for virtual teaching and learning are network-related problems, such as audio virtual discrepancies, session interruptions because of unexpected network logging out and persistent buffering (Gause et al. 2022). The sudden switch to digital learning methods highlighted how important it was for nursing students to gain high levels of clinical competency through experiences previously gained in clinical settings and now need to be duplicated or enhanced with online learning (Haanes et al. 2024). This transition has presented both opportunities and challenges, particularly in maintaining the quality of clinical training in a virtual format. The public perception of virtual clinical training sometimes rates lower than traditional methods, so institutions must start initiatives to enhance engagement with virtual learning (Edgar et al. 2022). The economic feasibility, immersive quality and expandability of virtual reality-based learning create advancements in professional expertise across different sectors (Kim et al. 2024).

Clinical and healthcare education benefits from virtual training tools, which boost technical skills yet find challenges to teaching essential abilities such as empathy and adaptability (Alcañiz et al. 2018). Kim and Park (2024) reveal that virtual reality significantly improves clinical skills development among nursing trainees by rectifying traditional clinical practicum constraints. The newly qualified nurses’ transition to qualification was distinctive (Godbold et al. 2024). Newly qualified nurses who had worked as students during the initial wave of the pandemic were judged to be more qualified, alleviating some of the known consequences of transition (Godbold et al. 2024). Moreover, Crismon et al. (2021) state that the pandemic worsened the stress of moving from higher education institutions as student nurses to new positions as registered nurses. Registered nurses complained about a misalignment between their expectations and the nursing job, higher workloads and eliminating transition programmes (Crismon et al. 2021). Virtual clinical learning replacement experience was statistically meaningful, leading to increased confidence in ensuring patient safety, enhanced professional communication abilities and acknowledged support in the workplace (Ulmen et al. 2022). Ravik et al. (2023) reveal that newly qualified nurses experienced feelings of being overwhelmed because of a deficiency in confidence regarding the practical elements of nursing. Despite this, a paucity of literature explores newly registered nurses’ experiences of learning clinical skills virtually during COVID-19 and its impact on their transition into professional practice in the Tshwane District of the Gauteng province in South Africa.

### Aim

The study aimed to explore and describe newly registered nurses’ experiences of learning clinical skills virtually during COVID-19 and its impact on their transition into professional practice in the Tshwane District of the Gauteng province in South Africa.

## Objectives

### The objectives of the study were as follows

- To explore the newly registered nurses’ virtual experiences of learning clinical skills during COVID-19 and its impact on their transition into professional practice in the Tshwane District of the Gauteng province in South Africa.
- To describe the newly registered nurses’ experience registered nurses’ experiences of learning clinical skills virtually during COVID-19 and its impact on their transition into professional practice in the Tshwane District of the Gauteng province in South Africa.

## Research methods and design

### Study design

A qualitative exploratory, descriptive and contextual design was executed to explore and describe newly registered nurses’ experiences of learning clinical skills virtually during COVID-19 and its impact on their transition into professional practice in the Tshwane District of Gauteng province in South Africa. A qualitative approach explores how people make sense of their surroundings, experiences and understanding of a phenomenon (Polit & Beck 2020). The qualitative exploratory and contextual design thus allowed for an in-depth exploration and understanding of newly registered nurses’ experience regarding clinical skills taught virtually during COVID-19 in Gauteng.

### Study setting

The study was conducted in two public hospitals in the Gauteng province. The hospitals were coded A and B. Hospital A is a tertiary and/or academic hospital, and hospital B is a central hospital. Both these hospitals are situated in the Tshwane district. This location was chosen because it was practical, accessible and pertinent. These hospitals are tertiary and central, and they receive many newly registered nurses, which made it practical to find the target population. The participants were numerically coded from 01 to 12.

### Study population and sampling

The focused study population and sample size comprised 12 newly registered nurses who were 1st, 2nd, 3rd and 4th year students in 2020 and currently employed as registered nurses in the Tshwane district. Purposive and snowballing sampling were utilised. The researcher intentionally selected the category of participants that most effectively enhanced the study and subsequently added new participants based on referrals from those selected participants after noticing a low turnaround relying on purposive sampling alone (Polit & Beck 2020). The researcher interviewed 12 participants, and data collection continued until data saturation was achieved on 14 February 2025 when no new themes emerged from the interviews.

### Inclusion criteria

The inclusion criteria of this study were as follows:

- All student nurses between 1st and 4th year in 2020 and 2021.
- Newly registered nurses who were taught clinical skills virtually and employed full time in the selected hospitals.
- Newly registered nurses studied either at nursing colleges or universities in South Africa.

### Exclusion criteria

The exclusion criteria of this study were as follows:

- Newly registered nurses who did not consent were excluded from participating in this study.
- Newly registered nurses who are short term contracted at the selected hospitals.

### Data collection

Prospective participants were recruited from the hospitals where they work. The researcher received statistics of nurses who were employed between 2021 and 2024. The announcement was made through notices on the noticeboard and word of mouth for those who meet the criteria to avail themselves through the Hospital Clinical facilitator, WhatsApp and phone calls to the researcher. Data were collected from November 2024 to February 2025 using unstructured, face-to-face interviews of approximately 45 min each. The interviews included open-ended and follow-up questions in English, enabling nurses to share their experiences freely. Interviews took place at various venues that were convenient to the participants; some were interviewed in the wards and library. A voice recorder was used, and there was no research assistant. Interviews are the most effective means for collecting diverse, inclusive and unique experiences (Roulston & Choi 2018).

An interview guide was drawn based on the research question, the study objectives and a literature review (Sokhela, Nokes & Orton 2024). The central question posed was: *What are your experiences of learning clinical skills virtually during COVID-19 and its impact on your transition into professional practice?* The researcher approached newly registered nurses, explaining the aim of the study. Subsequently, those who agreed to participate were asked to sign an informed consent form.

### Data analysis

Thematic steps were employed to analyse data. Thematic analysis is a method where researchers systematically organise and analyse complex data (Clarke & Braun 2013). These steps were applied: (1) Familiarise yourself with the data. Verbatim transcripts were read several times so that the researcher could be immersed in the data for understanding and interpretation. (2) Generating initial codes, data were condensed into meaning units consisting of words, sentences and chunks of text, which contained aspects related to each other. (3) Searching for themes, these meaning units were reduced and labelled with a code. Codes were sorted into categories and abstracted into themes. (4) Reviewing potential themes, the researcher agreed on the themes that emerged from the data. (5) Defining and naming themes, the independent coder confirmed themes and subthemes (Sokhela et al. 2024).

### Trustworthiness

Trustworthiness was achieved by applying Lincoln and Guba’s framework, which guarantees research credibility, dependability, confirmability, transferability and authenticity (Polit & Beck 2020). Credibility was achieved by conducting interviews until data saturation, engaging participants for 45 min, recording interviews and validating transcripts with the research supervisor. Dependability was achieved through the code, co-code method of analysis, where data were coded over an extended period to ensure consistency in coding (Sokhela et al. 2024). An independent coder was appointed to confirm data analysis until a consensus was reached; this ensured confirmability. Transferability was accomplished by providing detailed descriptions of the newly registered nurses’ experiences and contexts, making their meanings clear to the readers. The study underwent an authenticity check by the supervisors, examination board and external examiner to ensure that the data represented the sample under investigation. Thus, the design, methodology and conclusions were confirmed to be relevant to the research question correctly and without bias.

### Ethical considerations

Ethics approval was provided by the Faculty of Health Sciences Research Committee at the University of Pretoria under ref no. 303/2024. The National Health Research Database (NHRD) authorised permission via the ethics committees from the selected hospitals ref no: GP_202408_119. All audio recordings and interview transcripts were kept on a computer secured by a password known solely to the researcher. Participation was entirely voluntary, and informed consent was acquired from all participants, which included permission to be recorded during the interview. The data collected were kept by the researcher and accessible only to the research team. The Nursing Management (Clinical Facilitators) for both facilities were the gatekeepers.

## Results

### Demographic characteristics of registered nurses

The participants comprised 10 females and 02 males newly registered nurses. Table 1 gives the characteristics of the study participants.

**TABLE 1:** Demographic characteristics of the participants.

Participant	Age (years)	Gender	Highest qualification	Year Group in 2020	Discipline currently working
A-01	23	Male	Bachelor of Nursing Science	1st	Gynaecology ward
A-02	23	Female	Bachelor of Nursing Science	1st	Emergency department
A-03	22	Female	Bachelor of Nursing and Midwifery	1st	Orthopaedic ward
A-04	25	Female	Bachelor of Nursing Science	1st	Emergency department
A-05	33	Female	Bachelor of Nursing Science	4th	Intensive Care Unit
A-06	27	Female	Bachelor of Nursing Science	1st	Labour ward
B-07	22	Female	Bachelor of Nursing Science	1st	Neurosurgery
B-08	27	Female	Bachelor of Technology in Nursing Science	4th	Labour ward
B-09	24	Female	Bachelor’s degree	4th	Gynaecology ward
B-10	26	Female	Diploma in General Nursing and Midwifery	2nd	Labour ward
B-11	34	Female	Bachelor of Nursing Science and Art	3rd	Labour ward
B-12	40	Male	Bachelor of Technology in Nursing Science	3rd	Emergency department

### Themes and subthemes

From the data analysis, three themes emerged: (1) the impact of online learning on confidence in clinical practice, (2) the challenges of transitioning from online learning to practical application and (3) recommendations for enhancing online learning in nursing education. The themes are indicated in Table 2.

**TABLE 2:** Themes and subthemes that emerged from data analysis.

Themes	Subthemes
1. Impact of virtual learning on confidence in clinical practice	1.1 Confidence in basic nursing skills 1.2 Lack of confidence in advanced procedures 1.3 Confidence gained through virtual learning 1.4 Confidence gained through workplace experience
2. Challenges of transitioning from virtual learning to practical application	2.1 Lack of practical experience before hospital placement 2.2 Discrepancy between virtual learning and real-life application 2.3 Extended learning curve to integrate skills into practice
3. Recommendations for enhancing virtual learning in nursing education	3.1 The need for incorporating clinical virtual realities in nursing curriculum 3.2 The importance of practical, in-person training and skill demonstrations in nursing education 3.3 A hybrid learning approach for future success 3.4 Desire for continued engagement and resource accessibility

#### Theme 1: Impact of virtual learning on confidence in clinical practice (Table 2)

The study revealed that clinical virtual learning has affected participants’ confidence in clinical practice. This theme was justified with subthemes: confidence in Basic Nursing Skills, a Lack of Confidence in Advanced Procedures, Confidence gained through virtual learning and Confidence Gained Through Workplace Experience.

**Subtheme 1.1: Confidence in basic nursing skills:** In this study, participants who were mainly in the first year in 2020 indicated that they were taught various basic nursing skills such as hand wash, bed-making and several other skills virtually through Blackboard and YouTube. These participants indicated that they did not experience difficulties when transitioning from learning these skills virtually during COVID-19 into practice because they had an opportunity to learn about these skill in 2nd, 3rd or 4th year of their studies while still at the university before joining the practice:

‘I had confidence in performing hand washing, turning the patient, and bed-making when I was appointed as a registered nurse in this hospital because I had an opportunity to learn them in real life when I was still a student at the university.’ (B-07, 22, Female, Bachelor of Nursing Science)

‘I have learned and I was confident. So, I was able to apply most of the skills that I gained in first and second year to the workplace or to the work setting.’ (A-02, 23, Female, Bachelor of Nursing Science)

**Subtheme 1.2: A lack of confidence in advanced procedures:** The study revealed that registered nurses who were in their advanced stages of training and were taught advanced clinical skills virtually, such as performing an episiotomy, drawing blood for arterial blood gas and insertion of an intravenous line using blackboard and watching YouTube. The participants of this study indicated that they lacked confidence in performing those skills after being appointed as registered nurses. Although these registered nurses lacked confidence in performing these skills, they felt that some of the health care professionals were praising them that they can work independently and were left alone with expected vaginal delivery, insertion of intravenous (IV) lines and monitoring a patient admitted in the intensive critical care (ICU) unit without supervision by experienced supervisors:

‘I do not know about others, but I was at the final level, and I had no confidence in performing an episiotomy because I did not have an opportunity to learn that skill practically before I was appointed as a registered nurse. I only learned it online.’ (B-09, 24, Female, Bachelor’s degree)

‘I did lack confidence in performing skills in certain areas when I was appointed here, and that is when I knew that I had to start afresh and learn as if I am a student again …’ (A-05, 33, Female, Bachelor of Nursing Science)

‘I was placed in this hospital with four new community registered nurses. The staff in that hospital said we were terrific, even though I did not think so. They just left us to deliver two to three women in labour on our own, insert IV lines. Some of my colleagues were allowed to nurse a patient on a ventilator in the ICU.’ (B-12, 40, Male, Bachelor of Technology in Nursing Science)

‘Learning to draw blood for arterial blood gas is not enough to do it virtually alone. I want to go to the skills lab and touch at least the mannequin, but even on that, I want to touch a real patient to feel competent and confident.’ (B-08, 27, Female, Bachelor of Technology in Nursing Science)

**Subtheme 1.3: Confidence gained through virtual learning:** The study revealed that registered nurses gained confidence. In addition, there was an unstable network in certain areas as nurses were learning from home as students. Some students came from rural areas and others from townships, both of which negatively impacted their learning experiences. Even those from urban areas faced challenges because of network congestion and connectivity issues. Nevertheless, despite these hardships, access to recorded lessons and instructional videos provided a valuable learning resource that helped boost their confidence. Interviewees had the following to say:

‘Excellently so, I am what I am today because I have completed my qualification virtually. I am very confident. I execute my duties very well.’ (B-12, 40, Male, Bachelor of Technology in Nursing Science)

‘So, I had more options to watch how other people do it. I think the only gap is that some of the videos were practices that were not done here in South Africa, so the gap would be that maybe the medication is not the same, but they use that side, where there are still procedures that are done differently. It was perfect for me. I am a virtual learner, so it worked perfectly for me. It worked best for me personally.’ (B-07, 22, Female, Bachelor of Nursing Science)

**Subtheme 1.4: Confidence gained through workplace experience:** These study’s findings revealed that registered nurses gained confidence through workplace experience. Nurses mentioned that they took steps to ensure they get workplace exposure. They indicated that they had used community service (Comm Serve) to boast their confidence in performing clinical skills among patients to the point of sacrificing their time to rest:

‘I would not say I was confident when I was appointed here as a registered nurse. However, I tried using most of my comms to boast my confidence and learn how to care for my patients effectively.’ (B-11, 34, Female, Bachelor of Nursing Science and Art)

‘So, I decided not to take leave, especially over the festive season 2020. I decided to work, go the extra mile, and spend most of my time in the hospital. I utilised the time to learn and gain confidence to know what to do when left with the patient.’ (A-05, 33, Female, Bachelor of Nursing Science)

‘Comm serve really helped me to gain confidence and learn how to perform the skills among my patients practically, and based on that, I became very confident. Even in the medical outpatient department [*MOPD*], I performed well because I was exposed to clinical practice during my comm service. Even now, in the emergency department, I am the person who is trusted. In maternity, I would be left to deliver; the only time I was assisted with the delivery was when it was complicated.’ (B-12, 40, Male, Bachelor of Technology in Nursing Science)

#### Theme 2: Challenges of transitioning from virtual learning to practical application (Table 2)

The study discovered that registered nurses had challenges transitioning from virtual learning to practical application. This theme was justified with subthemes: Lack of Practical Experience Before Hospital Placement, Discrepancy Between Virtual Learning and Real-Life Application and Extended Learning Curve to Integrate Skills into Practice.

**Subtheme 2.1: A lack of practical experience before hospital placement:** The study revealed that lack of practical experience prior to hospital placement negatively impacted nurses’ practical application of skills learned virtually. Registered nurses also indicated that those who trained after the pandemic had 4 years of training to be exposed to clinical practice. This study found this to be disturbing for nurses and creating anxiety when faced with the reality of applying specific skills:

‘My clinical experiences now, to be honest, I can say that it was challenging when I was in the practical or hospital setting. At first … whatever I saw online was not something I did practically, so I never really got a chance to practice before I could go to the hospital.’ (A-06, 27, Female, Bachelor of Nursing Science)

‘I would say that when we graduated, I did feel a little bit that our exposure to the clinical environment was way less than maybe as with the groups still in school because they had the full four years to do all the skills. Even when learning anatomy, we learned it from pictures. To learn anatomy on an atlas where the veins are blue and the arteries are red, it is not what you would see in practice.’ (A-04, 25, Female, Bachelor of Nursing Science)

‘Then it catches up with you when you now must look after a patient. Then you are like, my skills are not so sharp because I do not have much exposure.’ (A-05, 33, Female, Bachelor of Nursing Science)

**Subtheme 2.2: Discrepancy between learning and real-life application:** This study revealed discrepancies between learning and real-life application. Registered nurses had to apply their critical thinking skills to know what to and what not to learn from the virtual material provided to them. The study participants were also worried that the videos they were watching online could teach them about treatment modalities that we do not have in South African health institutions. Registered nurses also had difficulties in simulating clinical skills at home, where it is highly impossible to recreate a hospital environment, thus leading to ethical concerns, especially when recording the video using their family members, friends and relatives:

‘Because when I go to campus or hospital settings, it was a thing that I had to do those skills on the actual person, which was different compared to virtual … let us say I was practising the skills on my friends or relatives.’ (A-01, 23, Male, Bachelor of Nursing Science)

‘I remember because when I had to do the Glasgow scale assessment, I used my mother as a patient. I had to record her, take a video while doing the assessment, and I was concerned about breached confidentiality …’ (A-06, 27, Female, Bachelor of Nursing Science)

‘So, I had more options to watch how other people use the skills, and I think the only gap was that some of the videos were practices that were not done in South Africa. For example, the gap would be that the medication used in the video may not be the same as that in South Africa. So, where procedures are still done, you have your own textbooks or guidelines to reference, so you can see what to take and what not to take from the video.’ (B-07, 22, Female, Bachelor of Nursing Science)

**Subtheme 2.3: Extended learning curve to integrate skills into practice:** The study revealed that registered nurses had an extended learning curve to integrate skills into practice. Registered nurses revealed that they had to adopt an attitude of life-long learning to enhance their knowledge and competence. Some facilities filled in the gap by encouraging nurses to identify where they lacked or felt unsure, and in-service training was arranged to teach registered nurses appointed after the COVID-19 pandemic:

‘[*O*]ver the past year, I would say that maybe that is where I tried to catch up, learn those skills, integrate what I learned and apply it in the practical setting. It took a little bit longer than the normal year that I would have learned it and grasped it.’ (A-04, 25, Female, Bachelor of Nursing Science)

‘When I started working, I knew I had to start afresh… Be a student again and learn; luckily, the department staff were supportive, so they knew the challenges, and they would ask you to identify areas where you felt like you are not so sure and guide you on that.’ (A-05, 33, Female, Bachelor of Nursing Science)

#### Theme 3: Recommendations for enhancing virtual learning in nursing education (Table 2)

This theme presents suggestions by different registered nurses on how to enhance virtual learning in Nursing Education. It was justified with subthemes: the need for Incorporating Clinical Virtual Reality into the Nursing Curriculum, the Importance of Practical, In-Person Training and Skill Demonstrations, a Hybrid Learning Approach for Future Success and the Desire for Continued Engagement and Resource Accessibility.

**Subtheme 3.1: The need for incorporating clinical virtual realities in nursing curriculum:** The study’s findings revealed that there is a need for incorporating clinical virtual realities in Nursing Curriculum by teaching student nurses technological tools, i.e. computers, how to record videos and share them with their lectures in preparedness for other similar pandemics in the future. Registered nurses justified this by indicating that some were from high school, had never touched a computer, did not know how to use it, and were suddenly expected to record themselves by making videos and sending them online to their lecturers for assessment:

‘I feel like there should be some sort of training when it comes to using, navigating these technology tools because imagine you are coming straight from high school, you did not do Computer Applications Technology [*CAT*] … now you are expected to just navigate everything on your own, record videos, send them to your lecturer for assessment.’ (A-02, 23, Female, Bachelor of Nursing Science)

‘I want to believe that there is a variety of virtual learning tools, I was only exposed to videos and images. However, I think it will be more efficient and effective to promote clinical virtual learning. Student nurses need to be trained to enhance their competence and skills in performing and applying the skills they have learned virtually to real patients.’ (B-08, 27, Female, Bachelor of Technology in Nursing Science)

**Subtheme 3.2: The importance of practical, in-person training and skill demonstrations in nursing education:** The study’s findings revealed that registered nurses shared a different view about the importance of practical, in-person training and skill demonstrations in nursing education. In this subtheme, registered nurses had different views. Some felt they did not need in-person clinical training because clinical virtual reality during COVID-19 was sufficient. In addition, they were taught by young lecturers who could navigate clinical virtual reality, which led to creative engagements with students. On the other hand, some felt there was a need for in-person skills demonstration in nursing education. They do not need clinical virtual realities at all because they negatively impacted them when they joined the workforce as registered nurses:

‘I would not recommend virtual learning or online learning. Especially for the skills you must perform on patients because that is not working well for us as nurses, especially when we had to perform the skill in real life.’ (A-01, 23, Male, Bachelor of Nursing Science)

‘I think we should not entirely cancel this virtual learning; it has its good. I am not saying we should solemnly stick to it, but I am not saying we must cancel it.’ (B-07, 22, Female, Bachelor of Nursing Science)

‘[*E*]verything. So, I do feel like it did disadvantage us because now we could not get to go to skills laboratory or simulations and, you know, get to practice the skills, go to the hospital and do the skills on the patients. So, I feel like it was very difficult for us.’ (A-02, 23, Female, Bachelor of Nursing Science)

‘She would go to the skills lab, open the projector, and show us. Alone in the skills lab. We were at home separately. Skills lab was well-resourced regarding these practical skills, presenting all the necessary tools, and we were fine by then. It was more effective than in-person.’ (B-07, 22, Female, Bachelor of Nursing Science)

**Subtheme 3.3: A hybrid learning approach for future success:** The study revealed that registered nurses viewed hybrid teaching and learning methods as essential for the future success of nursing education. The hybrid learning approach could be good, where some students engage in class face to face while others take part remotely and have the lecturer deliver lessons to both groups simultaneously:

‘It can be done by exposing students to all learning methods, being virtual learning, familiarising them with the technological tools, going to hospital as soon as they start the learning in the nursing.’ (B-11, 34, Female, Bachelor of Nursing Science and Art)

‘Using both face-to-face and virtual is very helpful, and it would be helpful to students and prepare them enough before practising as registered nurses; I believe they will be competent.’ (B-07, 22, Female, Bachelor of Nursing Science)

‘And then they can teach students a skill online, for example, about a pelvic exam, demonstrate pelvic exam in simulation laboratory… things like that I think would help.’ (A-03, 22, Female, Bachelor of Nursing and Midwifery)

**Subtheme 3.4: Desire for continued engagement and resource accessibility:** This study revealed that nurses desire continued engagement through developing short courses that can be used to bridge the clinical knowledge gap during COVID-19. They also indicated that Nursing Education Institutions could have allowed them to have access to online resources for a longer period to be able to bridge the gap between clinical virtual reality and practice:

‘So yes, it would be nice if the universities could have allowed us access to the videos developed by lecturers for a longer period so that we can refer back to them if we feel stuck in clinical practice as registered nurses.’ (B-07, 22, Female, Bachelor of Nursing Science)

‘Basic short courses could have helped with certain skills. The skills they know we missed would have to be communication between the nurses and nursing education institutions to engage them more through short courses.’ (A-05, 33, Female, Bachelor of Nursing Science)

## Discussion

The study explored and described registered nurses’ experiences of learning clinical skills virtually during COVID-19 and its impact on their transition into professional practice in the Tshwane District of the Gauteng province in South Africa. The study revealed that registered nurses did not face challenges from acquiring basic nursing skills virtually during COVID-19 to applying them in practice. They had the chance to learn these skills at a more advanced level while still attending the university prior to joining the workforce. The findings are supported by Padilha et al. (2019) that clinical virtual simulation enhances both immediate knowledge retention and long-term knowledge retention, it further increases the satisfaction level regarding learning experience for nursing students. Matlhaba and Khunou (2023) also indicated that the favourable learning experience might be associated with the self-confidence of practising procedures in a more welcoming environment.

This study also discovered that registered nurses felt less confident executing advanced clinical skills, e.g., suturing an episiotomy or inserting an IV line, as they transitioned to new nursing roles. Similarly, Godbold et al. (2024) revealed that the pandemic exaggerated students’ transition to qualified nurses. In support of this, a lack of transition programmes, greater workloads and a discrepancy between their expectations and the nursing role led to heightened stress for nurses transitioning from education to new nursing positions (Crismon et al. 2021).

The study revealed that registered nurses gained confidence through workplace experience because they used the time they were doing community service to enhance confidence in executing clinical skills with patients to the extent of sacrificing their time for rest. Some participants lacked confidence through virtual learning because there were no accessible videos shared by their lecturers to revise the clinical procedures. Alrashidi et al. (2023) support these findings, stating that when nursing students participate in clinical simulation training, their self-confidence levels increase, and they believe they can carry out comparable procedures on actual patients.

Wiesner (2023) revealed that the absence of realism, the capacity to engage with multiple patients, and the challenges in utilising technology were disadvantaged features of the virtual simulation for nurses. Thus, several nurses felt that the virtual simulation did not aid them in preparing them for professional practice. Similarly, this study revealed that registered nurses deemed the transition from virtual learning to practical application troubling and caused anxiety when confronting the necessity to implement specific on real patients.

This study discovered discrepancies between learning and real-life application because registered nurses needed to apply their critical thinking abilities in determining what to learn and what not to learn. Registered nurses were concerned that the videos they viewed virtually were not always instructing them about treatment modalities available in South Africa. Unsworth et al. (2016) investigated and identified the discrepancies after simulation sessions. Similar to the findings of this study, it was discovered that the scenarios caused some inconsistency regarding normal and abnormal physiology and common medications and their effects. Debriefing sessions primarily involved students being asked to reflect on and discuss their observations and actions regarding the patient’s condition. Therefore, it is important to have debrief sessions after virtual learning where opportunities for further development of this learning strategy can be addressed.

It was discovered that registered nurses had to extend the learning curve to integrate skills into practice, meaning they needed to embrace a mindset of continuous learning to improve their knowledge and skills. Many clinical settings lack adequate training materials, technology and supervision, especially those with minimal budgets. As a result, student nurses have limited clinical experience and practice chances (Salifu et al. 2019). Some facilities encouraged participants to pinpoint areas where they felt uncertain, and in-service training was organised to train them post-COVID-19 pandemic. Similarly, Martin, Conlon and Bowe (2021) emphasise the necessity of transitioning from theoretical scenarios to more engaging case studies grounded in actual environments and utilising real-time information.

South Africa is a nation characterised by significant socio-economic disparities and exceptionally elevated rates of poverty and unemployment (Khumalo 2021). Thus, this study revealed the need to incorporate clinical virtual realities in the nursing curriculum to make this teaching and learning strategy resourceful and impactful. Mashau and Nyawo (2021) found that university students need to be mandated to attend the computer literacy module because there is a strong connection between attending computer-related modules and possessing computer literacy.

The study indicated that nurses seek ongoing engagement, access to resources and a hybrid learning approach for future success to enhance registered nurses’ skills. They valued recorded videos of lecturers demonstrating clinical procedures, which they often revisited, as face-to-face learning relies on memory for recalling techniques. Similarly, Burton (2022) discovered that nursing students felt that access to YouTube videos showcasing skill demonstrations would enhance their preparation for acquiring psychomotor nursing skills. In support of this finding, nurses continued their formal professional nursing education by participating in professional development relevant to their practice areas (Sokhela et al. 2024). Thus, every participant who accessed the YouTube video series before acquiring the skills in the laboratory viewed the videos and stated that it readied them more effectively to learn the intravenous skills (Burton 2022). To promote lifelong learning and address historical inequalities in nursing education, short learning programmes (SLP) and courses were advocated in South African Higher Education (Van Jaarsveldt & Joubert 2018). According to Van Jaarsveldt and Joubert (2018), SLP delivery is dynamic and offered in a higher education learning context, where nurses can benefit from quality education, alongside university amenities and academic support such as SANC Continuous Professional Development (CPD) activities that are currently piloted, where these competencies’ deficiencies would be addressed.

### Limitations

The results of this study cannot be generalised because it only explored registered nurses working in two public hospitals, which restricts the applicability of the findings in other settings. The researcher recognises potential bias from close involvement, but this was managed through careful participant selection, trust-building and rigorous analysis to ensure honest and expressive data.

### Recommendations

The study strongly recommends universities and nursing colleges develop a hybrid training programme for the newly registered nurses who joined practice during COVID-19 to engage them more in SLP for the skills gap in training. The training institutions must partner with the NDoH to ensure the success of hybrid training programmes and that virtual reality environments directly link clinical teaching with optimal patient care. Instead of relying on YouTube videos during pandemics, training institutions should invest in different software to enhance virtual reality. Another way to make virtual teaching and learning more successful is to include Computer Applications Technology (CAT) courses and virtual reality orientation programmes in the nursing curriculum to boost nurses’ confidence. Furthermore, research is required to investigate the knowledge, attitudes and practices of virtual teaching methods.

## Conclusion

In conclusion, this study revealed the dual impact of virtual clinical learning on newly registered nurses during the COVID-19 pandemic. While some nurses gained confidence and skills, transitioning from virtual learning to practical application presented challenges because of insufficient hands-on experience. The findings highlight the need for nursing education institutions to improve virtual learning environments and integrate practical experiences, ensuring graduates are well prepared for professional practice. By addressing these challenges, nursing programmes can better equip new nurses to meet the high expectations of health care facilities and deliver quality patient care.
